# Wind Sock Deformity in Rectal Atresia

**DOI:** 10.4103/1319-3767.48974

**Published:** 2009-04

**Authors:** Sayed M. V. Hosseini, Farhad Ghahramani, Alireza Shamsaeefar, Tannaz Razmi, Mohammad Zarenezhad

**Affiliations:** Department of Surgery, Hormozgan University of Medical Sciences, Bandar Abbas, Iran

**Keywords:** Anorectal malformation, neonatal obstruction, rectal atresia, wind sock deformity

## Abstract

Rectal atresia is a rare anorectal deformity. It usually presents with neonatal obstruction and it is often a complete membrane or severe stenosis. Windsock deformity has not been reported in rectal atresia especially, having been missed for 2 years. A 2-year-old girl reported only a severe constipation despite having a 1.5-cm anal canal in rectal examination with scanty discharge. She underwent loop colostomy and loopogram, which showed a wind sock deformity of rectum with mega colon. The patient underwent abdominoperineal pull-through with good result and follow-up. This is the first case of the wind sock deformity in rectal atresia being reported after 2 years of age.

Rectal atresia is a rare form of anorectal malformation and is usually seen in male patients.[1] The most common types are complete fibrous membrane or severe stenosis between distal rectum and anal canal. They occur in neonatal period with bowel obstruction and are usually treated with colostomy.[2] Our aim in this case report is to present a new deformity (wind sock) of rectal atresia as the first case in literature, which has been missed until 2 years of age.

## CASE REPORT

**Figure 1:**
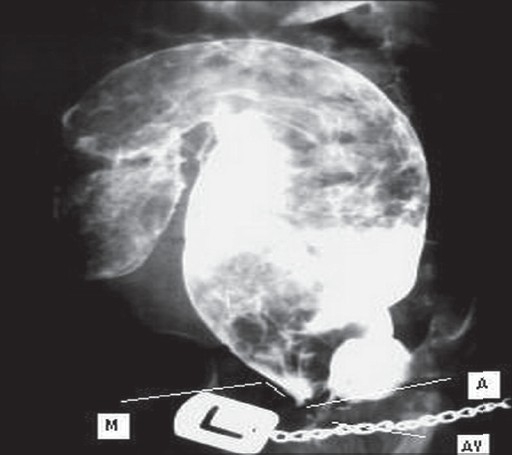
M-membrane,A-aperture,AV-anal verge preoperative colostogram

**Figure 2:**
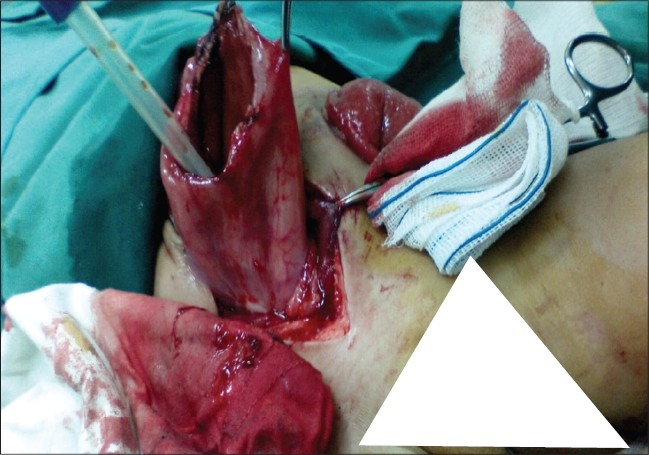
Rectal tube pulled through central aperture

**Figure 3:**
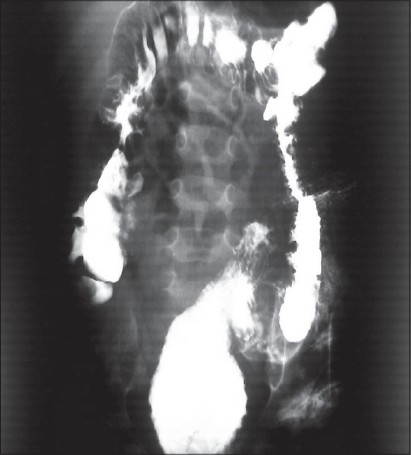
Post pull through cologram

A 2-year-old girl who was born at 38 weeks with a birth weight of 2300 g and admitted because of severe constipation, which started after birth. There were no medical or surgical problems in her history. In physical examination, she was a very thin girl with distended abdomen, mucosal pallor, and temporal wasting. Rectal examination revealed a 1.5-cm rectal pouch with scanty malodorous discharge. Abdomen had doughy consistency in favor of huge fecal impaction. She underwent a loop sigmoid colostomy with postoperative proximal and distal irrigation to remove the massive tarry stool. In the postoperative distal loopogram, a huge megarectum and a high wind sock rectal atresia (bulging membrane with tiny aperture) was found [Figure 1]. The patient's general condition and appetite had improved in the following two months. Thereafter, she underwent an abdominoperineal pull-through operation and protecting colostomy. In the second operation, a small clamp was passed through tiny central aperture via anal canal and a large rectal tube was easily pulled through the central hole, which made the membrane to be ruptured [Figure 2]. However, proximal resection of megacolon and pull-through of normal bowel was performed. We closed the colostomy after obtaining a normal distal loopogram [Figure 3] and the histology revealing normal ganglion cells in resected margin. The patient was discharged with good bowel function and continence.

## DISCUSSION

Rectal atresia with wind sock is an extremely rare defect reported in male patients in which the rectum may be totally or partially obstructed. The upper segment is usually a dilated rectum, whereas the lower portion is a small anal canal. It however has normal location and measures approximately 1–2cm deep. These defects occur in approximately 1% of the entire group of malformations, and they have all the necessary elements to be continent. Because they have a well-developed anal canal, they have normal sensation in the anorectum.[1,2]

These two portions are often separated by a thin complete membrane or by a dense portion of fibrous tissue, but an elongated membrane with central aperture has never been reported.[3] In our case, one theory is that the pressure of meconium caused necrosis and perforation of thin rectal membrane, which prevented bowel perforation, But, regarding her neonatal history, she had never developed any signs and symptoms of acute abdominal obstruction, and she had only scanty stool, whereupon she was treated for constipation, which implied to the natural existence of aperture. Because of stool consistency during neonatal and early infancy, her parents had hardly noticed the problem, but the changing diet caused the abdomen become gradually distended. However, the tiny aperture let gas and fluid to be passed, and the normal anal canal and inappropriate physical examination made the deformity be missed.

Many operative techniques have been used for the correction of rectal atresia. These techniques include abdominoperineal pull-through, single sacral approach, and abdominal and transanal approach.[4–6] Recently, laparoscopic-assisted pull-through has made the transanal pull-through much easier and safer by releasing of the rectum transabdominally.[7]

Each surgical technique has its own advantages and disadvantages. However, we have used abdominoperineal pull-through because a nonfunctioning megarectum should be resected, and the normal proximal colon was anastomosed above dentate line without disturbing the continence mechanism.

We used protecting colostomy because the distal portion was not adequately prepared and also a neurogenic function of distal portion was unknown.

## CONCLUSION

According to our knowledge, this is the first case of rectal atresia with wind sock configuration that was missed until 2 years of age. We recommend precise physical examination and workup of neonatal constipation to prevent missing of important etiologies. Regardless of the type of atresia and severity of chronic changes, adopting the least invasive option to preserve the continence is very important.
